# Research on Superconductivity in Multilayer ABC-Stacked Graphene

**DOI:** 10.3390/nano16080481

**Published:** 2026-04-17

**Authors:** Jun-Liang Wang, Jia-Xue Liang, Xiu-qing Wang

**Affiliations:** Physics and Electronic Information Institute, Inner Mongolia Minzu University, Tongliao 028043, China; 17320055273@163.com (J.-L.W.); 13643453290@163.com (J.-X.L.)

**Keywords:** superconductivity, external electric field, deformation potential model, linear combination operators, unitary transformation

## Abstract

Under the deformation potential model, the superconducting phenomenon in ABC-stacked multilayer graphene under a vertical electric field is investigated using linear combination operators and unitary transformation methods. Through the deformation potential model applied to a linear continuous medium, the effect of the external electric field is converted into the deformation potential energy of the crystal. Deformation potential phonons (LA phonons) act as propagators, generating electron–electron interactions. As the electric field increases, the ratio of the electric displacement vector to the dielectric function (D/ε) rises, leading to an increase in the electron ground-state energy, the opening of the band gap, and an enhancement of the attractive electron–electron interaction. With further increases in the external electric field, the deformation potential constant of the crystal ($D_{l}$) increases. When the phonon vibration frequency (ω) is around 8.5 THz, and the conditions are satisfied—where the wave vectors of different LA phonons are equal in magnitude and opposite in direction, and the electron spins are opposite—the attractive electron–electron interaction reaches its maximum ($H_{eff}$), resulting in the emergence of superconductivity. Our study also provides a new perspective for understanding the unique quantum properties—such as strong correlations, superconductivity, and ferromagnetism—in different stacking configurations like AB, ABC, and ABCA.

## 1. Introduction

With the discovery of graphene by K.S. Novoselov et al. in 2004 [1], researchers have shown great interest in this type of two-dimensional material that is bonded closely through covalent bonds on a plane and interacts through van der Waals forces across the plane. Graphene materials can have stacking patterns such as AA, ABA, ABC, etc. Recent experiments on Bernal bilayer (AB) and rhombohedral trilayer (ABC) graphene reveal cascades of correlated phases and superconductivity, thus providing a platform to study correlation effects in ultra-clean graphene-based systems [2,3,4,5,6,7,8,9]. Ample evidence show that the superconductivity detected in these non-twisted multilayer graphene is unconventional [10,11]. Up to now, there is still insufficient experimental evidence for the mechanism and pairing symmetry of these systems. Recent studies have shown that electron–infrared phonon coupling in ABC three-layer graphene may provide new insights into its superconductivity [12], as electron–phonon coupling may play a significant role.

Electron–phonon coupling is a fundamental interaction between basic excited states and plays a crucial role in various physical phenomena and condensed matter quantum phase transitions. For instance, electron–phonon coupling not only sets the inherent limit of electron mobility but also leads to extremely low thermal conductivity [13,14,15,16]. It also lays the foundation for charge density waves [17,18], electronic hydrodynamics [18,19,20], superfluidity [21,22,23], and superconductivity [24,25,26]. The unitary transformation (LLP) method proposed by Lee, Low, and Pines in 1952 has been widely used in dealing with electron–phonon interaction problems [27,28,29,30]. This study is based on the deformation potential model under the variational theory and uses the linear combination and unitary transformation methods to calculate the influence of electron–phonon interaction on the superconductivity in ABC-stacked graphene, providing a new perspective for understanding its special quantum properties (such as strong correlation, superconductivity, and ferromagnetism).

## 2. Materials and Methods

**Figure 1:**
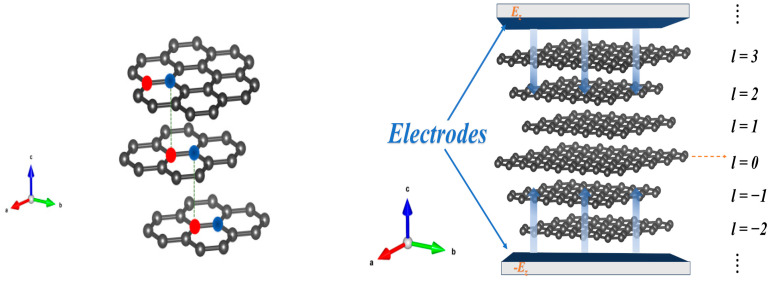
Multilayer ABC-stacked graphene with an applied vertical electric field.

Considering a multilayer ABC-stacked graphene structure under the application of a vertical electric field, by assuming the electric field strength is $E_{z}$ (V/m), perpendicular to the graphene plane, with an interlayer spacing of *d* (approximately 0.335 nm for graphene layers), and by taking the electric potential of the geometric center layer as zero as shown in Figure 1, the potential energy of the l-th layer (l=1,2,…,N) can be expressed as:

(1) $$U_{l}=e\frac{D}{\varepsilon }dl$$

where D is the electric displacement vector and ε is the dielectric function. Under the action of the external electric field, the interlayer distance changes, leading to crystal deformation. The external potential is thereby transformed into the crystal’s deformation potential energy, and variations in the deformation potential primarily affect the strength of electron–phonon interaction. In a two-dimensional system, where electrons interact with deformation potential phonons, specifically longitudinal acoustic (LA) phonons, the Hamiltonian of this 2D system can be written as [29,30]:

(2) $$H=\frac{p^{2}}{2m}+\sum_{q}\hbar sqa_{q}^{+}a_{q}+\sum_{q}V_{q}e^{iq\cdot r}a_{q}+h\cdot c-e\frac{D}{\varepsilon }dN$$

(3) $$V_{q}=i{\left[\frac{4\pi \alpha \hbar^{3}sq}{mA}\right]}^{\frac{1}{2}}$$

(4) $$\alpha =\frac{m{D_{l}}^{2}}{8\pi \rho \hbar^{2}s^{2}}$$

(5) $$\omega =\sqrt{\omega_{0}^{2}+{\Delta \omega }^{2}}$$

(6) $${\omega (LB}_{N,N-j})={\omega (LB}_{bulk})\sin\left[\frac{j\pi }{2N}\right]\;\;\;\;j=1,2,3\ldots ,N-1$$

(7) $${\omega (LB}_{bulk})=\frac{1}{\pi c}\sqrt{\frac{\alpha_{0}^{⊥}}{\mu }}$$

(8) ω=s·q

where m represent the center mass; $D_{l}$ is the deformation potential constant of the crystal; q is the 2D wave vector of an acoustic phonon; $a_{q}^{+}$ and $a_{q}^{+}$ are the creation and the annihilation operators of an acoustic phonon of wave vector q; α is the coupling parameters of the acoustic phonon with electron; ρ and A are the face density and the area of the crystals; s is the surface sound velocity of the crystals; $\omega (LB_{N,N-j})$ is LB mode frequency; ${\omega (LB}_{bulk})$ is the vibration frequency of the breathing mode of the bulk material; c is the speed of light; μ is the mass of atoms per unit area of a single two-dimensional material; $\alpha_{0}^{⊥}$ is the interlayer interatomic coupling constant of respiratory energy in a plane-external region; $\omega_{0}$ represents the phonon vibration frequency within a single-layer two-dimensional material layer; Δω represents the frequency of the LB model, which is the coupling frequency of interlayer van der Waals interactions; and ω is the phonon mode frequency in the high frequency layer. For multilayer graphene [31,32], the relationship between the number of layers and phonon frequency satisfies Equations (5) and (7).

In the following formulas, $b_{j}^{+}$ and $b_{j}$ are the creation and annihilation operators, respectively, and λ is a variational parameter [29,30].

(9) $$P_{j}={\left(\frac{m\hbar \lambda }{2}\right)}^{\frac{1}{2}}\left(b_{j}+b_{j}^{+}\right)$$

(10) $$r_{j}={\left(\frac{\hbar }{2m\lambda }\right)}^{\frac{1}{2}}\left(b_{j}-b_{j}^{+}\right)\;\;\;\;\;\;\;\;\;\;\;\;\;j=x,y$$

By substituting Equations (3)–(10) into Equation (2), the unitary transformation is performed:

(11) $$U_{1}=exq\left(-iB\sum_{q}a_{q}^{+}a_{q}q\cdot r\right)$$

(12) $$U_{2}=exp\left(\sum_{q}a_{q}^{+}f_{q-}a_{q}f_{q}^{*}\right)$$

In the above equations, $f_{q}$ are variational parameters; B represents the coupling strength parameter by Huybrechts; B=1 represents weak coupling; and B=0 represents strong coupling [33]. Then, Equation (2) can be written as:

(13) $$U_{2}^{-1}U_{1}^{-1}HU_{1}U_{2}=H^{\prime }=H_{0}+H_{1}$$

(14) $$\begin{array}{cc} H_{0}=\frac{\hbar \lambda }{4}\sum_{j}(b_{j}b_{j} & +b_{j}^{+}b_{j}^{+}+2b_{j}^{+}b_{j})+\frac{\hbar \lambda }{2}+\sum_{q}\left\{\hbar sq+\frac{\hbar^{2}q^{2}}{2m}\right\}\left(a_{q}+f_{q}\right)\left(a_{q}^{+}+f_{q}^{*}\right)\\ & \;-\frac{B\hbar }{m}{\left(\frac{m\hbar \lambda }{2}\right)}^{\frac{1}{2}}\sum_{q}\sum_{j}\left(a_{q}+f_{q}\right)\left(a_{q}^{+}+f_{q}^{*}\right)\left(b_{j}+b_{j}^{+}\right)q_{j}\\ & \;+\sum_{q}[V_{q}\left(a_{q}+f_{q}\right)e^{-(1-B)\frac{\hbar q^{2}}{4m\lambda }}e^{-(1-B){\left(\frac{\hbar }{2m\lambda }\right)}^{\frac{1}{2}}\Sigma_{j}q_{j}b_{j}^{+}}e^{-(1-B){\left(\frac{\hbar }{2m\lambda }\right)}^{\frac{1}{2}}\Sigma_{j}q_{j}b_{j}}\\ & \;+h\cdot c]-e\frac{D}{\varepsilon }dN \end{array}$$

(15) $$\begin{array}{lll} H_{1} & =\sum_{q^{\prime }\neq q}\frac{{B^{2}\hbar }^{2}}{2m} & \left[\left(a_{q}^{+}+f_{q}^{*}\right)\left(a_{q^{\prime }}^{+}+f_{q^{\prime }}^{*}\right)\left(a_{q}+f_{q}\right)\left(a_{q^{\prime }}+f_{q^{\prime }}\right)\right]\cdot q^{\prime }\cdot q\\ & =\sum_{q^{\prime }\neq q}\frac{{B^{2}\hbar }^{2}}{2m} & [a_{q}a_{q^{\prime }}a_{q}^{+}a_{q^{\prime }}^{+}+a_{q}^{+}a_{q^{\prime }}^{+}a_{q}f_{q^{\prime }}+a_{q}^{+}a_{q^{\prime }}^{+}a_{q^{\prime }}f_{q}+a_{q}^{+}a_{q^{\prime }}^{+}f_{q}f_{q^{\prime }}^{*}\\ & & +a_{q}^{+}a_{q}a_{q^{\prime }}f_{q^{\prime }}^{*}+a_{q}^{+}a_{q}f_{q^{\prime }}f_{q^{\prime }}^{*}+a_{q}^{+}a_{q^{\prime }}f_{q}f_{q^{\prime }}^{*}+a_{q}^{+}f_{q^{\prime }}f_{q}f_{q^{\prime }}^{*}\\ & & +a_{q^{\prime }}^{+}a_{q}a_{q^{\prime }}f_{q}^{*}+a_{q^{\prime }}^{+}a_{q}{f_{q^{\prime }}f}_{q}^{*}+a_{q^{\prime }}^{+}a_{q^{\prime }}f_{q}f_{q}^{*}+a_{q^{\prime }}^{+}f_{q}^{*}f_{q}f_{q^{\prime }}\\ & & +a_{q}a_{q^{\prime }}f_{q}^{*}f_{q^{\prime }}^{*}+a_{q}f_{q^{\prime }}f_{q^{\prime }}^{*}f_{q}^{*}+a_{q^{\prime }}f_{q}f_{q}^{*}f_{q^{\prime }}^{*}+f_{q}f_{q}^{*}f_{q^{\prime }}f_{q^{\prime }}^{*}]\cdot q^{\prime }\cdot q \end{array}$$

The ground-state wave function of the system is given by $\Phi =\phi \left(\rho \right)\left|0\right\rangle$, where $\phi \left(\rho \right)$ describes the wave function describing the motion of an electron and $\left|0\right\rangle$ is a zero phonons state, which satisfies:

(16) $$b_{j}|0\rangle =a_{k}|0\rangle =0$$

The upper limit of the ground-state energy is obtained by minimizing the expectational value λ.

(17) $$E\left(\lambda \right)=\left\langle \Phi \right|H^{\prime }\left|\Phi \right\rangle =\left\langle \phi \left(\rho \right)\right|F\left(\lambda \right)\left|\phi \left(\rho \right)\right\rangle$$

(18) $$H_{eff}=\;min\;F\left(\lambda \right)$$

In the above equations, $H_{eff}$ denotes the effective Hamiltonian of this two-dimensional system. By substituting Equations (14) and (16) into Equation (17) and (18), as well as considering the weak coupling limit (B=1), we can obtain the ground-state energy of the two-dimensional system:

(19) $$E_{0}=\frac{\hbar \lambda }{2}-e\frac{D}{\varepsilon }dN+\sum_{q}\left[V_{q}f_{q}+V_{q}^{*}f_{q}^{*}\right]+\sum_{q}{\left|f_{q}\right|}^{2}\left\{\hbar sq+\frac{\hbar^{2}q^{2}}{2m}\right\}$$

(20) $$\frac{\partial E_{0}}{\partial f_{q}}=0$$

(21) $$f_{q}=-\frac{V_{q}^{*}}{\hbar sq+\frac{\hbar^{2}k^{2}}{2m}}$$

(22) $$E_{0}=\frac{\hbar \lambda }{2}-e\frac{D}{\varepsilon }dN-\sum_{q}\frac{{\left|V_{q}\right|}^{2}}{\hbar sq+\frac{\hbar^{2}k^{2}}{2m}}$$

By replacing $\sum_{q}$ with $\frac{A}{4\pi^{2}}\int_{0}^{\infty }\int_{0}^{2\pi }qdqd\varphi$, the following formula could be obtained:

(23) $$E_{0}=\frac{\hbar \lambda }{2}-e\frac{D}{\varepsilon }dN-\frac{mD_{l}^{2}}{2\pi \rho s\hbar }Ln\left(1+\frac{\hbar q_{m}}{2ms}\right)$$

where $q_{m}$ is Debye wave vector.

**Figure 2:**
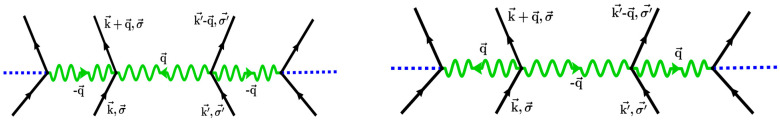
(**a**) Represents the Feynman diagram showing the propagation of q phonons and the interaction between electrons. (**b**) Represents the Feynman diagram for the propagation of −q phonons and the interaction between electrons.

In the Hamiltonian $H_{1}$, in the l-th layer, the part of the electron–electron interaction resulting from the propagation of LA phonons as shown in Figure 2 is:

(24) $$H_{e-e}=\sum_{q^{\prime }\neq q}\frac{{B^{2}\hbar }^{2}}{2m}\left[a_{q}^{+}a_{q}a_{q^{\prime }}^{+}a_{q}f_{q^{\prime }}+a_{q}^{+}a_{q^{\prime }}^{+}a_{q^{\prime }}f_{q}{+a}_{q}^{+}a_{q}a_{q^{\prime }}f_{q^{\prime }}^{*}+a_{q^{\prime }}^{+}a_{q}a_{q^{\prime }}f_{q}^{*}\right]\cdot q\cdot q^{\prime }C_{k_{l\sigma }}^{+}C_{k_{l\sigma }}$$

When B = 1, corresponding to a weakly coupled regime between electrons and LA phonons in the crystal, an attractive interaction emerges if $q\cdot q^{\prime }$ is negative and the electron spins are opposite. Once this attraction reaches its maximum at $q=-q^{\prime }$, the formation of Cooper pairs will produce flat bands, corresponding to the superconducting phenomenon:

(25) $$\begin{array}{c} H_{eff}=\sum_{q}\frac{\left\langle f|H_{ep}|m\rangle \langle m|H_{ep}|i\right\rangle }{E_{i}-E_{m}}\\ =-\sum_{q}{\left|f_{q}\right|}^{2}\frac{\hbar^{2}q^{2}}{m}\{\frac{\langle f|a_{-q}C_{k-q}^{+}C_{k}|m_{1}\rangle \left\langle m_{1}\right|a_{-q}^{+}c_{k^{\prime }+q}^{+}c_{k^{\prime }}|i\rangle }{\varepsilon_{k^{\prime }}-\varepsilon_{k^{\prime }+q}-\hbar \omega_{-q}}+\\ \frac{\left\langle f|a_{q}c_{k^{\prime }+q}^{+}c_{k^{\prime }}\left|m_{2}\right\rangle \left\langle m_{2}\right|a_{q}^{+}C_{k-q}^{+}C_{k}|i\right\rangle }{\varepsilon_{k}-\varepsilon_{k-q^{-}}\hbar \omega_{q}}\} \end{array}$$

Eliminating the repetition when calculating k and $k^{\prime }$:

(26) $$H_{eff}=-\frac{1}{2}\sum_{q}{\left|f_{q}\right|}^{2}\frac{\hbar^{2}q^{2}}{m}\left\{\frac{2\hbar \omega_{q}}{{\left(\varepsilon_{k}-\varepsilon_{k+q}\right)}^{2}-{\left(\hbar \omega_{q}\right)}^{2}}\right\}$$

Summing up and converting to integration yields:

(27) $$H_{eff}=-\frac{4D_{l}^{2}m^{3}}{\pi \rho \hbar^{4}}\left\{\frac{\hbar^{2}}{32m^{2}s^{2}}\cdot \left[Ln\left(1-\frac{2ms}{\hbar q_{m}}\right)-Ln\left(1+\frac{2ms}{\hbar q_{m}}\right)\right]-\frac{1}{{4\left(q_{m}+\frac{2ms}{\hbar }\right)}^{2}}+\frac{\hbar }{8ms}\cdot \frac{1}{q_{m}+\frac{2ms}{\hbar }}\right\}$$

## 3. Discussion and Results

**Figure 3:**
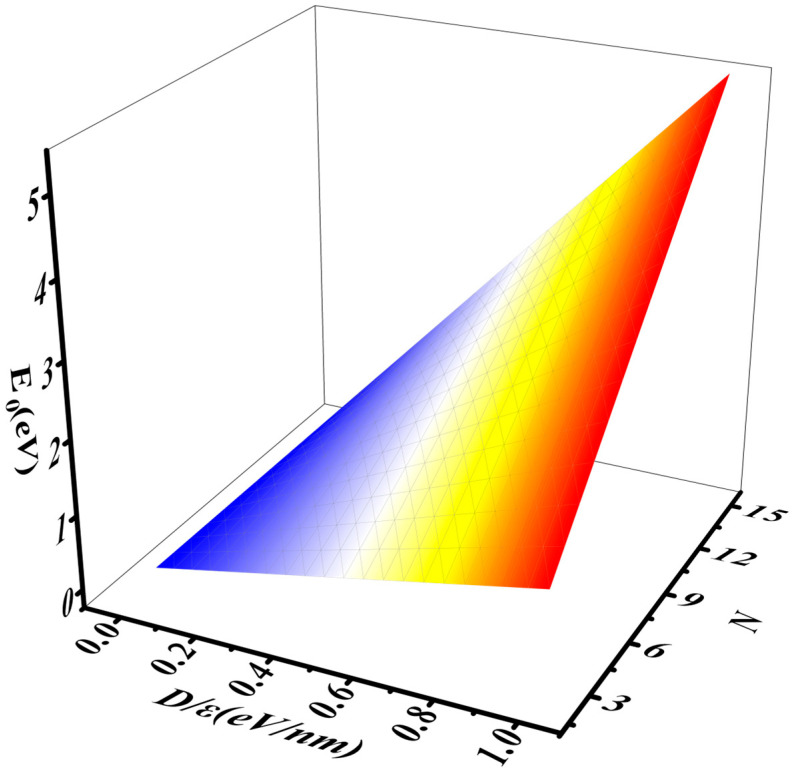
The relationship between $E_{0}$, D/ε, and N.

**Figure 4:**
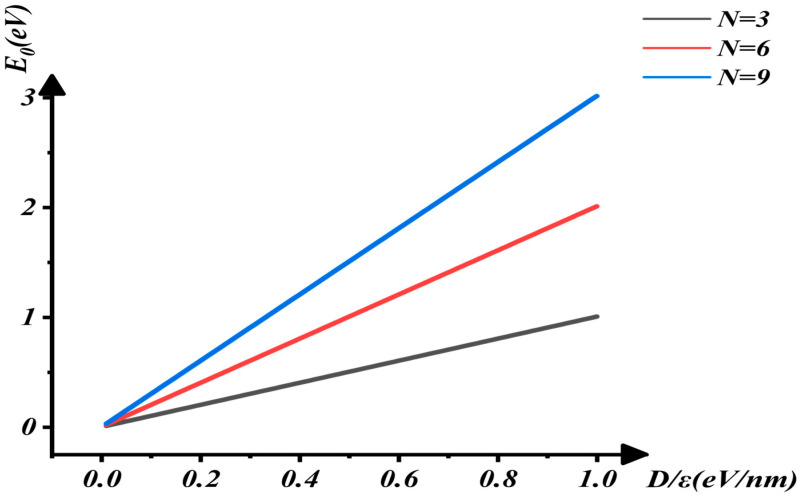
The interaction between $E_{0}$ and D/ε when N=3, 6, 9.

**Figure 5:**
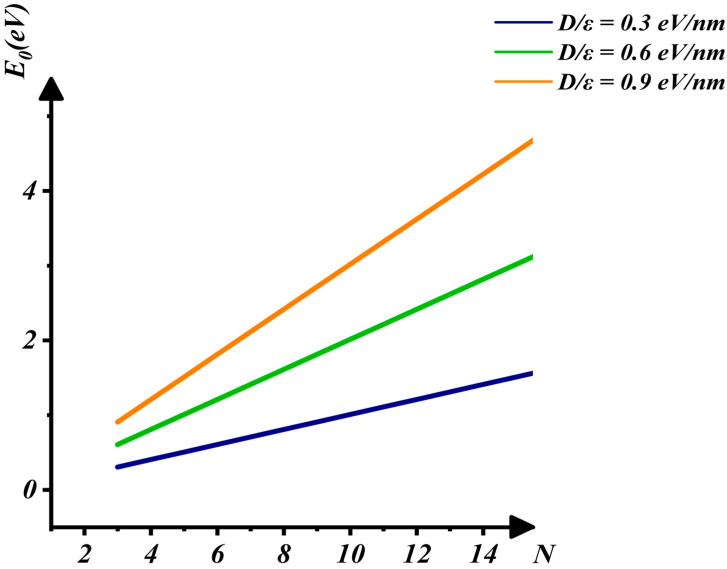
The interaction between $E_{0}$ and N when $\frac{D}{\varepsilon }=0.3,\;0.6,\;0.9$ eV/nm.

In this report, as shown in Figure 3, Figure 4 and Figure 5, it is demonstrated that E_0_ increases rapidly with the increases in D/ε and N. As the applied electric field increases, D=εE causes D/ε to rise, leading to an increase in the electron ground-state energy and the opening of the band gap. With an increase in the number of layers, the potential energy from the applied electric field also increases, thereby raising the electron ground-state energy. These variations are shown in Figure 4 and Figure 5. The characterization of the relationship between $E_{0}$ and D/ε when N=3, 6, 9 in Figure 4 indicates that $E_{0}$ increases rapidly as D/ε rises. Similarly, Figure 5 shows that $E_{0}$ increases rapidly as N increases.

**Figure 6:**
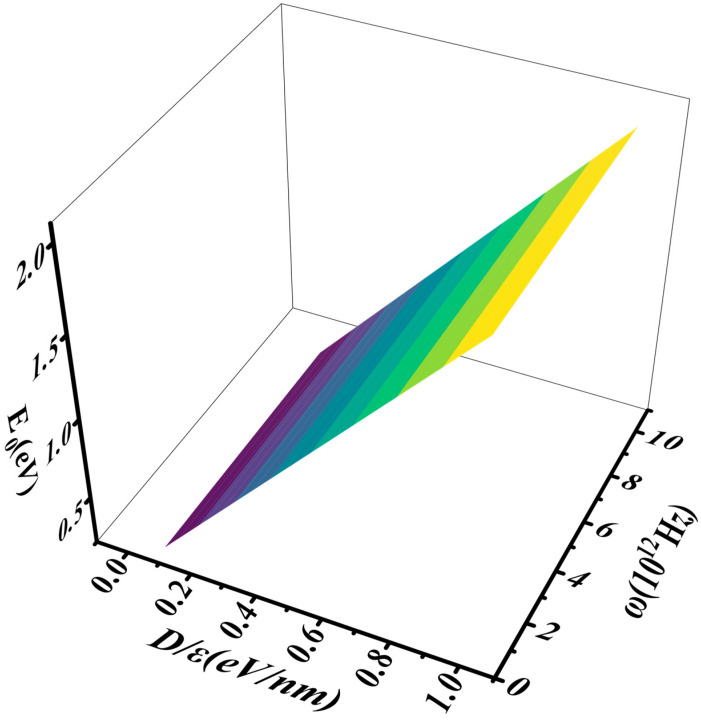
The interaction between $E_{0}$ and ω.

**Figure 7:**
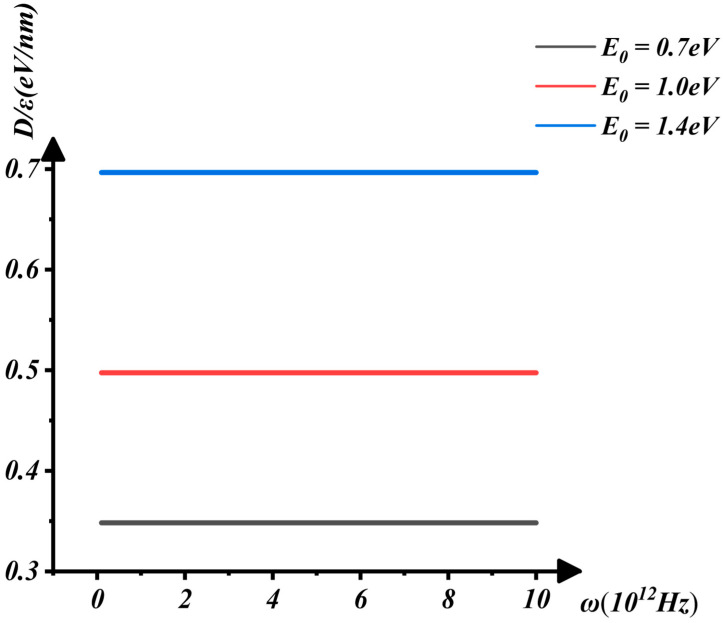
The interaction between D/ε and ω when $E_{0}=0.7,\;1.0,\;1.4$ eV.

Figure 6 and Figure 7 illustrate the variation of $E_{0}$ with ω and D/ε. Through Figure 6 and Figure 7, we observe that $E_{0}$ increases rapidly as D/ε rises. However, changes in D/ε have a minimal effect on the phonon vibration frequency but a significant impact on the electron ground-state energy.

**Figure 8:**
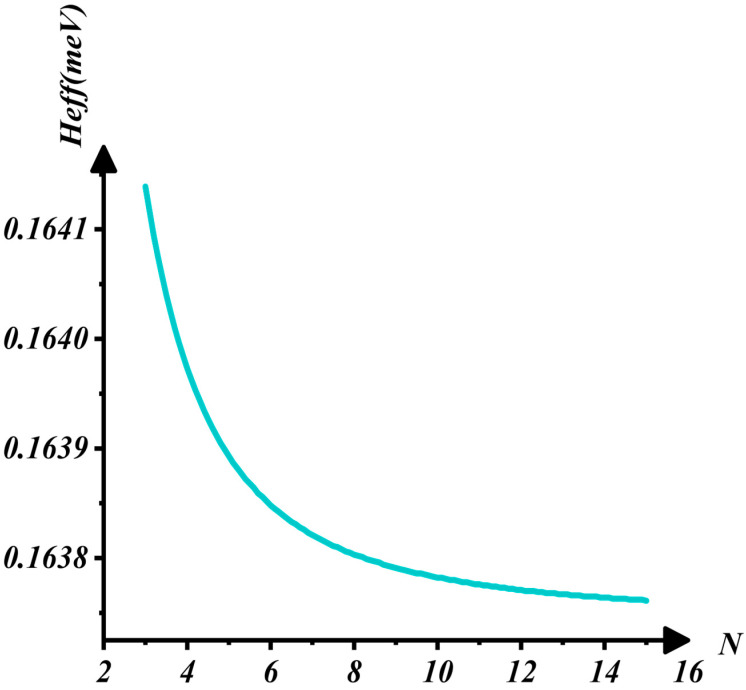
The interaction between $H_{eff}$ and N.

Figure 8 demonstrates that $H_{eff}$ decreases rapidly as N increases but stabilizes when N>8. This indicates that $H_{eff}$ is significantly influenced by N at lower layer numbers. According to the linear chain model, the elastic restoring force increases with N, leading to enhanced collective vibration of the entire layer, which strengthens the electron–phonon interaction and initially raises $\left|H_{eff}\right|$. However, as N increases, the interlayer van der Waals force weakens gradually, thereby reducing its contribution to the electron–phonon interaction. Consequently, when N reaches a threshold, such as N=8, $H_{eff}$ tends to saturate and approaches a constant value. This behavior suggests that while increasing the number of layers enhances the electron–phonon interaction, the effect is ultimately limited by quantum confinement.

**Figure 9:**
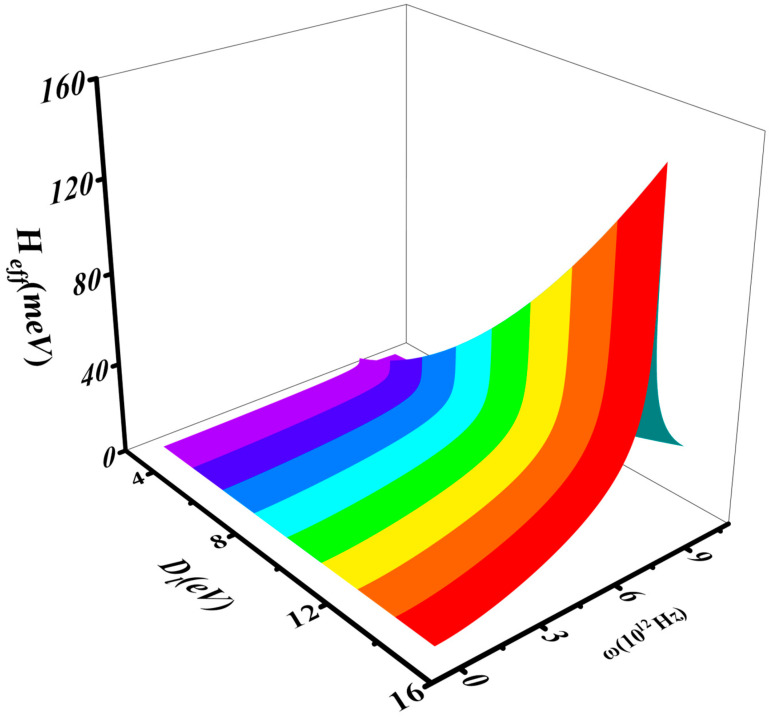
The interaction between $H_{eff}$, $D_{l},$ and ω.

**Figure 10:**
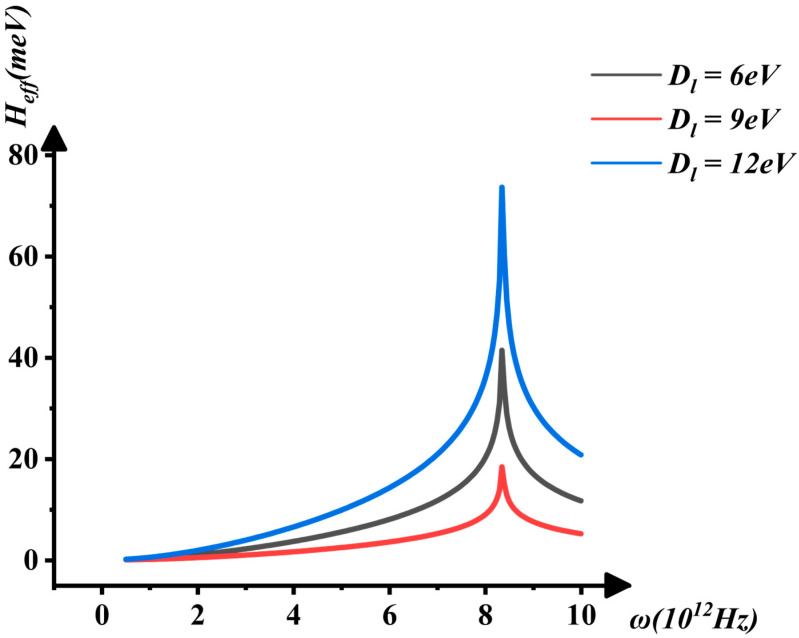
The interaction between $H_{eff}$ and ω when $D_{l}=6,\;9,\;12\;eV$.

Figure 9 and Figure 10 show the dependence of $H_{eff}$ on ω and the deformation potential constant $D_{l}$. As $D_{l}$ increases, the phonon vibration frequency ω rises, but it drops sharply when ω>8.5 THz, as shown in Figure 10. Near this abrupt transition point, the crystal’s properties undergo a change, leading to the emergence of superconductivity, which is attributed to the interaction between electrons and infrared phonons. This observation aligns with experimental results [34]. A possible explanation is that, as the electric field increases, the ground-state energy of electrons with opposite spins rises, resulting in the opening of the band gap. When the attractive interaction between electrons reaches its maximum, satisfying the conditions for superconductivity, Cooper pairs form and give rise to the superconducting state.

## 4. Conclusions

In this work, the deformation potential model is applied to describe the external electric field, while the linear chain model is employed to characterize the interlayer van der Waals interactions. Using the methods of linear combination and unitary transformation, we investigate the influence of electron–electron interactions, mediated by LA phonons as propagators, on the superconductivity of ABC-stacked multilayer graphene under a vertically applied electric field. The main findings are as follows:

1. As the external electric field increases, the ratio of the electric displacement vector to the dielectric function (*D*/*ε*) rises. This leads to an increase in the electron ground-state energy, the opening of the band gap, and an enhancement of the attractive electron-electron interaction. When the interaction reaches the binding energy of Cooper pairs, superconductivity emerges.
2. Under the applied electric field, the electron ground-state energy increases with the number of layers, whereas the attractive potential of the electron–electron interaction decreases. For the same external electric field, ABC-stacked systems with fewer layers exhibit a stronger influence on the attractive interaction, making it easier to form Cooper pairs and induce superconductivity compared to systems with more layers.
3. This work also provides a new perspective for understanding the special quantum properties (such as strong correlation, superconductivity, and ferromagnetism) of different stacking patterns, such as AB and ABCA.

## Data Availability

This is a theoretical study based on analytical derivations. No experimental data were collected or generated. All mathematical results are fully reproducible from the equations and methods described in the main text.
